# Contemporary Philanthropy in the Spotlight: Pushing the Boundaries of Research on a Global and Contested Social Practice

**DOI:** 10.1007/s11266-021-00343-9

**Published:** 2021-03-25

**Authors:** Georg von Schnurbein, Marta Rey-Garcia, Michaela Neumayr

**Affiliations:** 1 Center for Philanthropy Studies (CEPS), University of Basel, Basel, Switzerland; 2 Department of Business, University of A Coruña, Campus de Elviña s/n, 15071 A Coruña, Spain; 3 Department of Management, WU - Vienna University of Economics and Business, Vienna, Austria

**Keywords:** Philanthropy, Contested concept, Power relations, Giving circles, Corporate foundations

## Abstract

This article is intended as the leading article in a special issue devoted to the achievements, limitations, opportunities and risks entailed in the research and practice of contemporary philanthropy. The article first characterizes philanthropy as a highly diverse and dynamic set of social practices that has only recently been subject to the systematic scrutiny of an emerging field of research, parallel to its rapid transformation and increased societal visibility. The main debates that emerged during the last two decades while researching the complexities of contemporary philanthropy are contextualized from the perspective of multiple disciplines; and the main foci for contentious conceptualizations and societal expectations explored. In this context, contributions of the special issues are summarized. Further avenues for pushing the boundaries of philanthropy research in ways inclusive of the dynamism, diversity, multi-disciplinarity and controversy that characterize the field, while at the same time providing meaningful answers to societal concerns about the potential and shortcomings of new philanthropic practices, are drawn.

## Introduction

Philanthropy is an ancient, complex and globally ubiquitous social practice that encompasses a highly diverse array of manifestations and has been traditionally subject to misconceptions and criticism. Voluntary action for the public good (Payton, 1988) adopts forms that vary widely over time and also across geographic, policy and cultural contexts as these may foster or, alternatively, hinder its development (Barman, 2017). It can be enacted by and benefit individuals (Bekkers & Wiepking, 2011), families (Feliu & Botero, 2016; Moody et al., 2011) or organizations (Gautier & Pache, 2015). It may be based on altruistic and other motivations; and be deployed through different tools and approaches and with various degrees of formalization. It may consist of monetary and/or non-monetary contributions (in-kind donations, volunteer time and/or expertise and other assets, blood and organ donations). It causes psychological, financial and social effects for donors, recipients and their respective institutional, sectoral and organizational contexts. However, it is interesting to note that despite broad variability of the phenomenon across time and geography, the “why” (the antecedents and motivations), “what” (the definition), “who” (the actors), “how” (individual and organizational behaviors) and “what for” (the effects) of philanthropy have been consistently subject to controversy throughout history (Moody & Breeze, 2016).

What is unprecedented about contemporary philanthropy is the speed of its evolution and its global societal visibility. Changes in the environment are rapidly reshaping philanthropic actors and their behaviors. If the ongoing effects of the COVID-19 pandemic are yet to be systematically assessed, two long-range environmental trends that have accelerated the pace of change of philanthropy in recent years should be mentioned. First, and related to shifts in institutional conditions, new behaviors and forms of organizing are emerging as a result of the continuous redesign of policies and legal or tax rules and regulations affecting philanthropy across countries. Some new behaviors respond to the need of philanthropic actors to collaborate cross-sector to harness new resources and relationships (e.g., partnerships including philanthropic, business and/or public actors); some new forms are far less institutionalized than foundations (as paradigm for a philanthropic institution). The latter include vehicles that remain relatively under-researched: from donor-advised funds to giving circles in the US; from sheltered foundations in France to *fonds hébergés* in Belgium (Eikenberry, 2006; Rey-Garcia, 2020). Secondly, as a result of digitization, philanthropic behaviors are being transformed at the micro- and meso-levels, from helping cost-effectively to recruit and engage volunteers through internet-connected devices (Medina, 2016), to democratizing giving through crowdfunding platforms and other online fundraising tools (Alegre & Moleskis, 2019).

In terms of visibility, the philanthropic initiatives by high profile living individuals—high-net-worth donors, “philanthrocapitalists” (Bishop & Green, 2008) or “philantrepreneurs” (Taylor et al., 2014)—businesses and global corporations in connection with current economic, social and environmental challenges (including global ones such as poverty or climate change) are widely attracting public attention. Their visibility has been infused by an unparalleled inter-generational transfer of wealth at the turn of the twenty-first century, coupled with the controversy generated by the adoption of business-like approaches to solving social problems and the globalization of family and corporate philanthropy (Bies & Kennedy, 2019; Bishop & Green, 2008; IUPUI Lilly Family School of Philanthropy, 2020; Rey-Garcia & Puig, 2013).

The point of departure of this special issue precisely consists of acknowledging that, as a result of the accelerated change and global visibility of philanthropic practices, their sources for legitimacy, financial accountability, political influence and (expectations for) public benefit outcomes have become, more than ever before, under the spotlight of public opinion. Contending societal expectations on the meaning and implications of philanthropy should accordingly inform and be embraced by the research agenda on the topic.

However, if any global phenomenon experiencing rapid change and public controversy is challenging to grasp from a scientific perspective, at least two additional barriers hamper the advance of philanthropy research. The first barrier lies in the difficulty in reaching conceptualizations that provide an inclusive understanding of the rich diversity of philanthropic actors and practices around the world and pave the way for future comparative studies. While research on individual philanthropy is skewed toward an understanding of this term as typical of affluent white men (Herzog et al., 2020), research on institutional philanthropy largely concentrates on the undertakings of endowed, grant-making foundations along the US standard (Toepler, 2018). Connected to this, the second barrier relates to the many hurdles involved in collecting and measuring evidence on the economic operations and social performance of philanthropy, as systematic data are scarce except for the US and a few other Western countries (for Europe see Hoolwerf and Schuyt, 2017; Wiepking, 2009); the same geographic locations that, not by chance, dominate the consolidated field of nonprofit studies.

It was precisely in this context that the special issue was initiated by members of the European Research Network on Philanthropy (ERNOP), a network that aims to advance, coordinate and promote excellence in philanthropic research. While ERNOP understands philanthropy as private, mainly voluntary contributions to public causes, it also acknowledges that philanthropy has very different meanings around the world and even across Europe, because of large variations in historical, social and legal backgrounds of philanthropy (ERNOP, 2021). The special issue aims at contributing to institutionalization of the emerging field of scholarly inquiry on philanthropy based on the two criteria that have guided the work of the network: interdisciplinarity and responsible research.

This article highlights the need to inclusively consider the scholarly debates on the many faces of philanthropy under the complementary lights of the diverse theories and concepts that are nurturing philanthropy research today. Multi-disciplinarity aids in better understanding the complexities and controversies surrounding the achievements and limitations of philanthropy when creating value for society. Additionally, ground is laid for the need to design philanthropy research that not only integrates the diversity of practices and disciplinary approaches, but also tries to provide answers to conflicting societal expectations about the significance and outcomes of philanthropy. Finally, conclusions about the potential opportunities and risks arising in the interface between academic and societal perspectives on contemporary philanthropy are drawn from individual contributions in the special issue.

## Approaching Current Debates on Contemporary Philanthropy from Diverse Disciplinary Backgrounds

Basically, the various theories and concepts linked to philanthropy today arise from four different streams of research: historical science, psychology, economics, and sociology. The historical perspective of philanthropy deals with the changes in the meaning of philanthropy from more religious mode to a political, a social and finally a fiduciary mode (Sulek, 2010a). It evolves from the philosophers of the antiquity, is suppressed in the medieval age by the religious concepts of charity or benevolence and is reinvented in the seventeenth century (Sulek, 2010b). The modern understanding of philanthropy develops in the nineteenth century with the rise of the leaders of the industrial revolution. The core aspects of the “scientific philanthropy” (Anheier & Leat, 2006) emerging in the nineteenth century are: the shift from local support to global activities; an expansion of causes beyond alms and basic needs to science, culture and education; the use of science and technology to tackle roots rather than symptoms of social problems and a delineation from the religious core of charity (von Schnurbein, 2015). Regardless of the respective understanding of philanthropy throughout history, philanthropy has always been accompanied by criticism and many of the arguments in today’s debate have been raised in former times. Perpetuity of foundations, the dominance of the founder’s will, and the injustice of the origin of great wealth have been criticized throughout the centuries (Hammack & Anheier, 2013; Prewitt, 2006; Reich, 2018).

The psychological research on philanthropy addresses motives and behavior of individuals. Giving is analyzed in relation to values, beliefs, attitudes and personal identity. Studies for instance show that people who are high in empathy, in emotional stability, in self-esteem, in locus of control or in moral development display higher levels of helping behavior (cf. Bekkers & Wiepking, 2011). Additionally, religion, but also psychological benefits are major intrinsic drivers for philanthropy (Pharoah, 2016). The most prominent psychological benefit is the feeling of “helpers high” (Luks, 1988) or a “warm glow” (Andreoni, 1990), which is used as an explanation for the non-altruistic motivation of philanthropy. However, recent psychological studies have questioned the intentions and motives of giving. In experimental designs, e.g., an extended dictator game with exit option on average 50% of the participants choose the exit option—violating both, the altruistic and the egoistic strategy (Cain et al., 2014). Dana et al. (2006) mention image concerns and the intention to avoid creating expectations as reasons why people rather give in instead of giving with dislike.

The final two disciplines, economics and sociology, analyze philanthropy on the individual, the organizational and the societal level. While the former is rooted in rationalist and individualist models, the latter is based on collectivist and normative approaches to the non-market sphere of society (Adloff, 2016). In the economic perspective, philanthropy on the individual level is included in the utility function, examining the costs and benefits of giving, thus extending the individual utility with receiving a good feeling (“warm glow”) or by integrating third-party utility as a general benefit. In sociological studies on the micro-level, the attitudes to and motives for philanthropy are discussed in relation to the individual’s position in society, i.e., their social, cultural and economic resources (Neumayr & Handy, 2019). As pure altruism is rare, philanthropy is mostly connected to reciprocity—or generalized reciprocity (Adloff, 2016). Individual giving is based on social norms and trust in the benevolence of others. The most important reason for people to give, is being asked (Bekkers & Wiepking, 2011).

On the organizational level, management research has focused on grant-making foundations as the gold standard for institutional philanthropy, especially on questions of governance (Romero-Merino & Garcia-Rodriguez, 2016), grant selection, performance in terms of fundraising and giving patterns (Koushyar et al., 2015) and impact measurement (Benjamin & Campbell, 2020). Research on the socio-economic performance, accountability and transparency of operating foundations (Rey-Garcia et al. 2018; Sanzo Pérez et al., 2017) has just emerged. However, other alternative forms of philanthropic organizing such as community foundations (Harrow et al., 2016), corporate foundations (Roza et al., 2020), giving circles (Eikenberry, 2006), or more entrepreneurial approaches (Kramer, 2010) have gained increased attention. Additionally, the question about the most efficient and effective way to manage philanthropy remains unsolved. From a sociological perspective, the power relation between donors and beneficiaries (be they individuals or organizations) has been analyzed for a long time (Ostrander & Schervish, 1990). Due to their financial potential, donors play a significant role in how nonprofit organizations operate, especially major donors and/or for certain areas of activity (Neumayr, 2015), with the result that upward rather than downward accountability is prioritized (Benjamin, 2010).

On the macro-level, the major question is the relationship between public welfare and private philanthropy, e.g., whether philanthropy is complementary, substitutional, or distinct (Anheier & Daly, 2007). Recent studies have revisited the widespread assumption that government expenditures reduce private donations (crowding-out) by disentangling aggregate measures to analyze whether government expenditure in a particular field crowds out or alternatively crowds in donations to the same, or instead, to other fields across countries, and to analyze consequences on the incidence and the level of donations (De Wit et al. 2018; Pennerstorfer & Neumayr, 2017). Connected to this is the increasing debate about taxation of philanthropy and different models of tax benefits (OECD, 2020). High tax exemptions for donors are criticized as unjust and not democratically justifiable (Reich, 2018). Finally, and connecting the meso- and macro-levels, a framework using the varieties of capitalism, welfare regimes and the social origins classifications has been proposed to advance comparative studies on foundations, that further examines their roles and performance in the proximity to the business sector and civil society (Anheier, 2018).

## Contemporary Philanthropy as Essentially Contested Concept

Parallel to the emergent institutionalization of scholarly debate on several levels of analysis and from different disciplines, visibility of philanthropy has increased tremendously in the past years. At no time in history, more money was given to charitable causes, and more foundations were created and more people were working in and for philanthropic organizations. Annual giving in the United States of America is at $449.64 billion, and in Europe it is estimated at EUR 87.5 billion (Hoolwerf & Schuyt, 2017; IUPUI Lilly Family School of Philanthropy, 2020). There are about 120,000 foundations in the U.S. and about 150,000 foundations in Europe. But philanthropy has also increased in other countries such as China or Russia, and regions such as South America or Africa. However, the percentage of giving on GDP remains stable in most countries.

Both, the dynamism and the increase in visibility have put philanthropy in the spotlight. From the perspective of societal expectations, recent shifts in public opinion and policy environments reflect conflicting views on the legitimacy of philanthropy and its influence on the political arena. One can find great stories about philanthropic support to specific causes such as natural or human catastrophes or the search for a vaccine against COVID-19. In contrast, recent books criticizing the philanthropy of the super-rich have fueled the debate on private wealth and public obligation. In Europe, this discussion was inflamed by the announcements of major donations after the blaze of Notre Dame in Paris in 2019. Additionally, on the policy level many countries have revised their laws on philanthropy in order to liberalize giving. But at the same time, space for civil society activities has narrowed in many countries (Anheier, 2017), a development that visibly culminated in the displacement of the Open Society Foundation from Budapest in 2019.

These contending views are mirrored to some extent in the current academic debate on the scope of philanthropy and its effects. A controversy that suggests that the need for conceptual clarification and systematic evidence on at least the “what” and the “what for” of philanthropy remains unfulfilled, despite recent advances in philanthropy research as a serious field of scholarly inquiry (Moody & Breeze, 2016). We use an inclusive definition of philanthropy as private, mainly voluntary contributions to public causes which builds on the open understanding by other researchers such as Payton (1988, p. 7), who defines philanthropy as “every voluntary action for the public good”, or Salamon (1992, p. 10), who states that philanthropy is “the private giving of time or valuables for public purposes.” While the former highlights the origin of philanthropy in the actions of an individual person (or a group of persons), the latter puts the emphasis on the philanthropic resources. Both aspects are important criteria for the political perception and handling of philanthropy, especially in terms of taxation, where tax exemptions are granted to individual donors and the amount of tax exemption is restricted to the measurability of the donations.

However, other researchers limit philanthropy to specific types of actors of activities. Most commonly, philanthropy is restricted to money donations (Harrow, 2010) or donations, bequests and foundations by wealthy people, only (Herzog et al., 2020). Given these various understandings of the scope and content of philanthropy, it can be described as an essentially contested concept (Daly, 2012; Payton & Moody, 2008; Van Til, 1990). Daly (2012) exemplifies the essentially contested nature of philanthropy based on Gallie’s (1956) approach highlighting the differences in the weighting and meaning of defining criteria. For instance, the voluntary character of philanthropy is questioned by researchers such as Schervish (1998) who argues that rather a sense of virtue and obligation is inherent to philanthropy, at least in the US context. Besides further criteria, Daly (2012) emphasizes the multi-dimensionality in the application of philanthropy through prefixes such as “catalytic” (Kramer, 2010), “strategic” (Frumkin, 2010), “venture” (Letts et al., 1997), or “impact” (Duncan, 2004), while others consider these mushrooming prefixes as an indicator for the increase in the importance/hype of the concept of philanthropy (Phillips & Jung, 2016).

More recently, the debate on philanthropy has turned into a controversy about the public value of philanthropy. Critical voices see philanthropy as the symptom of an unjust system that proliferates social inequalities (Giridharadas, 2018; McGoey, 2015; Reich, 2018). Giridharadas (2018) posits that philanthropy is build and used by the same elite power networks as in business and politics and that these networks exclude large portions of the people. Reich (2018) criticizes the non-democratic decision-making processes in philanthropy and the deprivation of public income for private preferences. The lack of accountability or responsiveness of foundations to their communities (McGinnis Johnson, 2016), to democratic norms (Reich, 2018), to the size and/or needs of marginalized groups (Kan et al. 2019), to social justice goals (Kohl-Arenas, 2015), or downward to beneficiaries (Rey-Garcia et al., 2017) has been highlighted. In contrast, other publications emphasize the positive public value of philanthropy, especially through approaches that oblige philanthropists to effective realization (Buchanan, 2019; Frumkin, 2010).

## The Contributions

In realm of these developments, we seek to offer in this special issue some conceptual and empirical foundations for further research on philanthropy that is inclusive of both the diversity of its manifestations and the multi-disciplinarity and dissent that guide their assessment and interpretation. Throughout the articles presented here, the described aspects of contestations, critics and appreciation, theoretical and empirical vagueness will become apparent. However, at the same time, the contributions offer conceptualizations and analysis that lay ground for a more profound and comprehensive debate, particularly regarding a broader but at the same time more nuanced understanding of philanthropy, regarding issues of inclusiveness, democracy, power relations and accountability.

The lack of understanding of philanthropic behavior from a global perspective is the initial point of the first article of the special issue, a conceptual paper by Wiepking (2021). She posits that macro-level studies that include philanthropy in all forms and across all geographic areas would improve the visibility of philanthropic contributions to society. To date, policy implications on philanthropic behavior, as well as economic, demographic or social changes are not consistently analyzed. In her article, three main barriers for a more profound of super-ordinated, contextual study of philanthropy are presented. The first barrier is the geographic orientation of research. It is highly concentrated on U.S., Australia and Western European countries. A major driver for this imbalance (besides the origin of the researchers) is the lack of data for other countries and regions. Another barrier is the frequent reduction in philanthropy to action by rich, white, old men. In this narrow sense, researching philanthropy on a macro-level seems like praising activities of only few individuals. Closely connected to this is the contestation of defining characters of philanthropy as discussed before. The dominating formal, instrumental understanding of philanthropy might not cover the full picture of philanthropic behavior on a global scale. As an answer to overcome these barriers, Wiepking (2021) tentatively suggests to replace philanthropy with generosity, because the term seems to have a more positive connotation. Additionally, there is a need to include and intensify research from other geographic areas and to provide better comparable data.

A path for further research that is inclusive of non-elite philanthropy is offered by Carboni and Eikenberry (2021). In their quantitative study on members and non-members of giving circles (GCs), they apply social capital theory to test donor identity and giving to historically marginalized groups. Bonding social capital develops based on donations to peer organizations, while bridging social capital is created by donation beyond the own group of reference. In contrast to the common connection of philanthropy to wealthy people, GCs represent a more “democratic” form of philanthropy, because the members of the GC participate in agenda-setting, decision-making and control of actions. Additionally, GCs may also lead to external democratic outcomes, such as donations to less favorite groups. Using the concept of identity, the authors show that all groups (GC members and non-members) tend to give to people with shared identity. However, bridging capital is more likely to be nurtured by GC members, e.g., they give to groups beyond their own identity. The authors conclude that GCs are a valuable tool to democratize philanthropy and strengthen social inclusion, as GCs are easy to set up and to run.

The seminal study by Ostrander and Schervish (1990) applied social relations theory to describe the power imbalance between donors and beneficiaries. In their contribution to this special issue, Oelberger and Shachter (2021) build on the assumption that foundations are private, powerful and relatively unrestricted. But they enlarge the study of the dominant role to transnational giving and focus on foundations that transfer funds to organizations in other countries. In recent years, many governments have passed laws that restrict the acceptance of funds from other countries for local nonprofits. The motivation for these confinements may be found in the attempt to preserve national sovereignty, and they stand vis-à-vis the efforts of foundations to support global solutions to complex environmental and social issues that do not end at national borders (e.g., climate change or human rights). Based on an analysis of all transnational grants of US foundations from 2000 to 2012, the study at hand deals with the influence of restrictive foreign law on foundation activity. The results show that restrictive laws generally have no influence on foreign activities of US foundations. To some extent, restrictive laws have even the opposite effect and seem to raise attention by foundations and funders. The authors discuss these findings in light of the power imbalance. With regards to the causes, the study removes fears that restrictive national laws will hinder the efforts for these causes. However, the findings support the fact that foundations feel no strong resistance when giving money to other countries. Thus, the authors call for further research on elite philanthropy and transnational donations.

Power relations lie also at the core of the study by Toepler and Abramson (2021) which analyzes government-foundation relationships, given the increase in formal partnerships between philanthropic institutions and state agencies in recent years. One of the novelties of their study consists of proposing a framework to understand partnerships between grant-making foundations and the government, informed by empirical evidence from the perspective of liaison officers in US federal agencies. In describing this relationship, the authors highlight four different roles of foundations: supplementation, substitution, innovation and social/policy change. The empirical findings confute one prominent argument of foundation literature, as government representatives do not expect foundations to be innovative, rather to join in co-creation and co-design of programs. Predominantly, the role of foundation was seen in supporting public services, thus accepting a subordinate function. These findings offer explanations for often mentioned tensions in the relationship between foundations managers and government representatives. The authors call for more research on partnership patterns in public-philanthropic partnerships.

It is one of the major reasons for current critics that philanthropy actors often are not hold accountable for the results of their actions. Two contributions suggest that accountability of philanthropic actors deserves particular research attention. The above-mentioned article by Oelberger and Shachter (2021) calls for academic debate on the extent to which US philanthropy would be advancing civil society and rights abroad with an unrestricted plutocratic power and, more generally, for increased research on transnational accountability and the influence of philanthropic actors on national sovereignty in foreign countries. Williamson et al. (2021) address a very specific type of philanthropic actors in their contribution. Public Ancillary Funds (PubAFs) are an Australian category of grant-making foundations that include different types of foundations such as corporate foundations, community foundations, or fundraising foundations. Authors concentrate on PubAFs that are in a significant, exclusive and close relationship (dyadic partnership) with another organization, e.g., a company, a church, or another nonprofit organization. Authors question whether or not the PubAF’s identity and accountability is influenced by this relationship. Results stem from a qualitative analysis with semi-structured interviews with representatives of PubAFs and show positive, neutral and negative consequences of the dyadic relationship. Advantageous are financial benefits, extended networks and risk mitigation, for instance. Complacent effects are mostly connected to differences in expertise or tasks. Resentments were reported for lack of visibility, limited distance between the dyadic partners, or missing transparency. Often, negative factors led to an increase in accountability. Key to good accountability is the leadership personnel and the standards applied. This study highlights the need for further research on factors influencing accountability of philanthropic organizations and how they can be managed.

Finally, in order to achieve a more inclusive understanding of philanthropy it is also necessary to highlight the special features and characteristics of the individual philanthropic forms. Through a systematic literature analysis of 80 publications covering 30 countries worldwide, Gehringer (2021) offers a comprehensive overview of corporate foundations. Corporate foundations are at the same time linked to the parent company and civil society, a feature that is often referred to as ‘hybrid.’ However, the existing literature rarely explains what is meant by hybridity of corporate foundations and how it is shown. The author identifies fifteen characteristics that differentiate corporate foundations in terms of establishment, organizational capabilities, purpose and outcomes. In terms of hybridity, the founding body, the underlying intentions, resources and impact of foundation activities are most important. Strategic hybridity means that corporate foundations blend societal and market forces to create advantages for both, company and society. Organizational hybridity describes the organizational design reflecting the alignment with the corporate founder and with the roots in the nonprofit sector. Finally, contextual hybridity contains the various influences of multiple constituents that affect the structures and operations of the corporate foundation.

## Conclusions

The essentially contested nature of philanthropy causes conceptual ambiguities and miscommunication and should be more recognized in theoretical studies and further developments of the topic. Articles in this special issue acknowledge this common ground and respond to the twofold need to reinforce conceptualization and analysis of philanthropy while responding to societal concerns about its undertakings, by suggesting further research that is inclusive along three dimensions.

First, this special issue sheds light on the barriers that hinder more solid conceptualizations of philanthropy and highlights the need for comparative studies that take into account diversity in the contextual conditions of individual philanthropy at multiple levels. Although the article by Wiepking (2021) responds to the call by Barman (2017) to focus attention on a macro-level in face of the predominance of micro-approaches in extant literature, truth is that multi-level analyses tend to be even more scarce than those at a macro-level (Liket & Simaens, 2015). Elucidating the institutional, sectoral and organizational conditions that underlie the successes and failures of philanthropic practices across different national contexts seems key to answer societal concerns about their impact on public affairs and global problems.

Second, contributions support the need to intensify inquiry on forms of philanthropy that have been subject to less intense research attention due to their geographic location, socio-demographic profile, relative novelty or organizational complexity. In contrast with the dominant focus on Western, elite, highly formalized or single-organization settings as foci for philanthropy research, this issue explores alternative forms that are characterized by more horizontal power dynamics (the case of giving circles, see Carboni & Eikenberry, 2021) and more complex forms of organizing. These forms require analytical tools that lie in between the macro- and meso-levels and theoretical approaches that can explain the behavior of philanthropic actors where different institutional logics coexist, due to hybridization and/or close interdependency with actors from the same or other sectors. This is both the case of corporate foundations (Gehringer, 2021) and partnerships between public agencies and foundations (Toepler & Abramson, 2021) or dyadic relationships between public ancillary funds and other organizations (Williamson et al., 2021).

Third and last, this issue draws scholarly attention toward the need to further cross-fertilize the multi-disciplinary approach to philanthropy with perspectives in the proximity of political science. The topics of power imbalances and accountability that are explored here (Oelberger & Shachter, 2021; Toepler & Abramson, 2021; Williamson et al. 2021) refer back to the idea of philanthropic practices and institutions as inherently political creatures (Anheier & Leat, 2006; Meyer et al. 2020). Not only they are shaped by public policies, but they may actively engage with public policy-making every time they select the public good causes or problems to fund, operate or advocate; endorse or adopt diagnoses and possible solutions to tackle them; select target beneficiaries of their activities or giving; or partner with other institutions, organizations or networks, intra- or cross-sector, to mobilize resources or relationships around shared agendas. The political nature of these choices holds regardless of the fact that those philanthropic practices or institutions may be tax subsidized. The more philanthropy shapes public discourse, ideas, values and policies, the more it becomes under the spotlight of public and scholarly scrutiny. In contrast with the risks that certain forms of elite philanthropy entail for democracy (Skocpol, 2016), this special issue suggests the need to take into account more inclusive definitions and alternative forms for approaching and organizing philanthropy that may entail opportunities for democratizing its research and its practice, both socially and geographically.
